# 新型手性杀虫剂唑虫酯异构体拆分及其在蔬菜中的分析方法建立

**DOI:** 10.3724/SP.J.1123.2022.01011

**Published:** 2022-07-08

**Authors:** Yan CHEN, Congling HUANG, Xunyuan JIANG, Zhiting CHEN, Gang WANG, Kai WAN, Xuemei TANG

**Affiliations:** 广东省农业科学院农业质量标准与监测技术研究所, 农业农村部农产品质量安全检测与评价重点实验室, 广东省农产品质量安全风险评估重点实验室, 国家农业检测基准实验室(农药残留), 广东 广州 510000; Institute of Quality Standard and Monitoring Technology for Agro-products of Guangdong Academy of Agricultural Sciences;Key Laboratory of Testing and Evaluation for Agro-product Safety and Quality, Ministry of Agriculture and Rural Affairs;Guangdong Provincial Key Laboratory of Quality & Safety Risk Assessment for Agro-products; National Benchmark Laboratory for Agricultural Testing (Pesticide Residues), Guangzhou 510000, China

**Keywords:** 高效液相色谱-串联质谱, 唑虫酯, 小白菜, 蕹菜, 手性分离, high performance liquid chromatography-tandem mass spectrometry (HPLC-MS/MS), pyraquinil, *Brassica rapa ssp. chinensis* L., *Ipomoea aquatica* Forsk, chiral separation

## Abstract

以全新手性杀虫剂唑虫酯为研究对象,通过筛选手性色谱柱和优化流动相比例,建立了唑虫酯及其氧化代谢物异构体的拆分方法,在此基础上开发利用高效液相色谱-串联质谱(HPLC-MS/MS)同时测定小白菜和蕹菜中唑虫酯及其氧化产物手性异构体的分析方法。以纤维素-三(3,5-二氯苯基氨基甲酸酯)共价键合手性柱(Chiral INC)(250 mm×4.6 mm, 5 μm)为分析柱,乙腈和2 mmol/L甲酸铵水溶液作为流动相进行梯度洗脱分离,在多反应监测负离子模式下进行检测,唑虫酯4个异构体分离度分别为1.63、2.83和1.74,唑虫酯氧化产物异构体分离度为5.82。通过衍生化的方法进一步确定出峰顺序为*RS*-唑虫酯、*SS*-唑虫酯、*RR*-唑虫酯、*SR*-唑虫酯、*S*-唑虫酯氧化产物和*R*-唑虫酯氧化产物。唑虫酯和其氧化产物的手性异构体分别在1.25~1250 μg/L和2.5~2500 μg/L范围内具有良好的线性关系,相关系数(*R*^2^)大于0.99。在蕹菜和小白菜样品中同时添加唑虫酯和唑虫酯氧化产物消旋体进行添加回收试验,添加水平为1、20、400 μg/kg(即唑虫酯异构体为0.25、5、100 μg/kg;唑虫酯氧化代谢产物异构体为0.5、10、200 μg/kg),回收率为72.6%~110.6%,相对标准偏差(RSD)均在9.4%以下,其中日内重复性的RSD在0.5%~9.4%之间;日间重复性的RSD在1.0%~8.6%之间,表明该方法具有良好的回收率和精密度。该研究可为唑虫酯这一新型手性农药的环境行为研究及后续质量控制、药效评价等提供相应的分析技术,为新农药开发应用提供有力的技术支撑。

## Abstract (en)

Pyraquinil (Pyr), with a new skeleton of pyrazolo[1,5-*a*]quinazoline fused heterocycle, is a new chiral insecticide independently developed by South China Agricultural University in 2017. In previous studies, we found that pyraquinil can effectively control the lepidopteran pest population on cruciferous crops. Remarkably, the insecticidal activity of pyraquinil was 64-fold better than that of fipronil against the fipronil-resistant *Plutella xylostella* field population. Pesticides with new active mechanisms should be developed in the future to cope with the development of resistance to *Plutella xylostella*. Therefore, pyraquinil with new active sites has the potential to be the main rotation variety for the control of *Plutella xylostella*. Thus, pyraquinil has a broad prospect for application in the future. However, a chiral separation and analysis method for pyraquinil and oxidation products using high performance liquid chromatography-tandem mass spectrometry (HPLC-MS/MS) has not been established yet.

Based on the physical and chemical properties of the new chiral pesticide pyraquinil, we screened chiral chromatographic columns and optimized the mobile phase ratio in this study. The separation and analysis methods for pyraquinil were developed based on the QuEChERS (quick, easy, cheap, effective, rugged, safe) system. Simultaneously, we also established oxidation metabolites. These methods were used for the simultaneous determination of the chiral isomers of pyraquinil and oxidation products in pakchoi (*Brassica rapa ssp. chinensis* L.) and water spinach (*Ipomoea aquatica* Forsk) via HPLC-MS/MS. The Chiral INC column (250 mm×4.6 mm, 5 μm) was used for separation. Ammonium formate aqueous solution (2 mmol/L) and acetonitrile were used as the mobile phases. The column temperature was 28 ℃. The injection volume was 1 μL, and the flow rate was 0.5 mL/min. MS analysis was performed using an electrospray ionization source in the negative and multiple reaction monitoring modes. We found that under the optimized conditions, the resolution of the four isomers of pyraquinil were 1.63, 2.83, and 1.74, respectively, and the resolution of the isomers of the pyraquinil oxidation product was 5.82, which achieved baseline separation. Then, the absolute configuration and peak order of pyraquinil and oxidation product isomers were determined by derivatization. The order of the peaks was *RS*-Pyr, *SS*-Pyr, *RR*-Pyr, *SR*-Pyr, *S*-Pyr+O, and *R*-Pyr+O. The purification conditions of sample pretreatment were optimized; 1 g (0.835 g MgSO_4_+0.150 g PSA+0.015 g GCB) was determined to be the optimal purification agent; and the average recoveries ranged from 80% to 110%. The chiral isomers of pyraquinil and oxidation products showed good linearity in the concentration range of 1.25 to 1250 μg/L and 2.5 to 2500 μg/L respectively. The square of the regression coefficient of the linear equation (*R*^2^) was greater than 0.99. The matrix effects of the pyraquinil and oxidation product isomers in pakchoi ranged from 6.1 to 30.6. In the water spinach, the matrix effect of the pyraquinil and oxidation product isomers were in the range of 0.7-26.8. The average recoveries of pyraquinil isomer at three spiked levels of 0.25, 5, 100 μg/kg in samples (pakchoi and water spinach) ranged from 90.2% to 110.6%. The oxidation product isomer average recoveries in samples (pakchoi and water spinach) spiked with 0.5, 10, 200 μg/kg ranged from 72.6% to 100.1%. Further, the relative standard deviations (RSDs) were 0.5%-9.4%. In water spinach, the intra-day and inter-day repeatability RSDs ranged from 0.5% to 8.7% and 1.0%to 8.6%, respectively. In pakchoi, the intra-day and inter-day repeatability RSDs ranged from 0.6% to 9.4% and 1.0% to 7.6%, respectively. These results indicate that the proposed method has satisfactory sensitivity, accuracy, and precision. This study can provide analytic technology for a novel chiral pesticide for environmental behavior studies, quality control, and pharmacodynamics evaluation, as well as significant technical support for the development and application of new pesticides.

在我国销售的化学农药中,手性农药在市场中占比达到40%,占据重要地位^[1,2]^。手性农药不同对映体在环境行为、生物活性、毒性等方面往往存在差异^[3⇓-5]^,而在评估手性农药对非靶标生物和生态环境造成的实际危害时,没有区分手性异构体差别,评估结果不够准确和全面^[6⇓⇓-9]^。因此,手性拆分是研究手性农药毒理毒性和环境选择性行为的前提和基础^[10⇓-12]^。

目前最广泛使用的手性农药对映体拆分分析方法是色谱法,其中常见的色谱法包括:气相色谱法、液相色谱法、超临界流体色谱法等^[13,14]^。而液相色谱法随着QuEChERS前处理方法的普及,具有分析时间较短、分离效率高等优点,适用更广泛^[15⇓⇓-18]^。Li等^[19]^利用高效液相色谱-串联质谱仪(HPLC-MS/MS)同时分离甲霜灵等6种手性杀菌剂,分离度在1.38~3.46之间。齐艳丽等^[20]^建立了手性超高效液相色谱-串联质谱检测小麦及其加工制品中腈菌唑对映体残留的分析方法。黄玉芬等^[21]^采用优化的QuEChERS样品前处理技术和HPLC-MS/MS,建立了同时测定土壤中3种手性杀菌剂对映体的分离分析方法。

唑虫酯(pyraquinil, Pyr)是华南农业大学徐汉虹课题组研发的一种具有吡唑并[1,5-*a*]喹唑啉稠合杂环骨架的全新手性杀虫剂,含有两个手性中心,4个手性异构体(见图1)。因其对抗性品系小菜蛾具有良好的防治效果^[22⇓⇓-25]^,且其在靶标上的作用位点有别于现有氨基丁酸(GABA)受体抑制剂杀虫剂,如狄氏剂、氟虫腈、阿维菌素等^[22]^,唑虫酯有望成为防治小菜蛾的主要轮换品种,具有广阔的市场前景。

**图 1:**
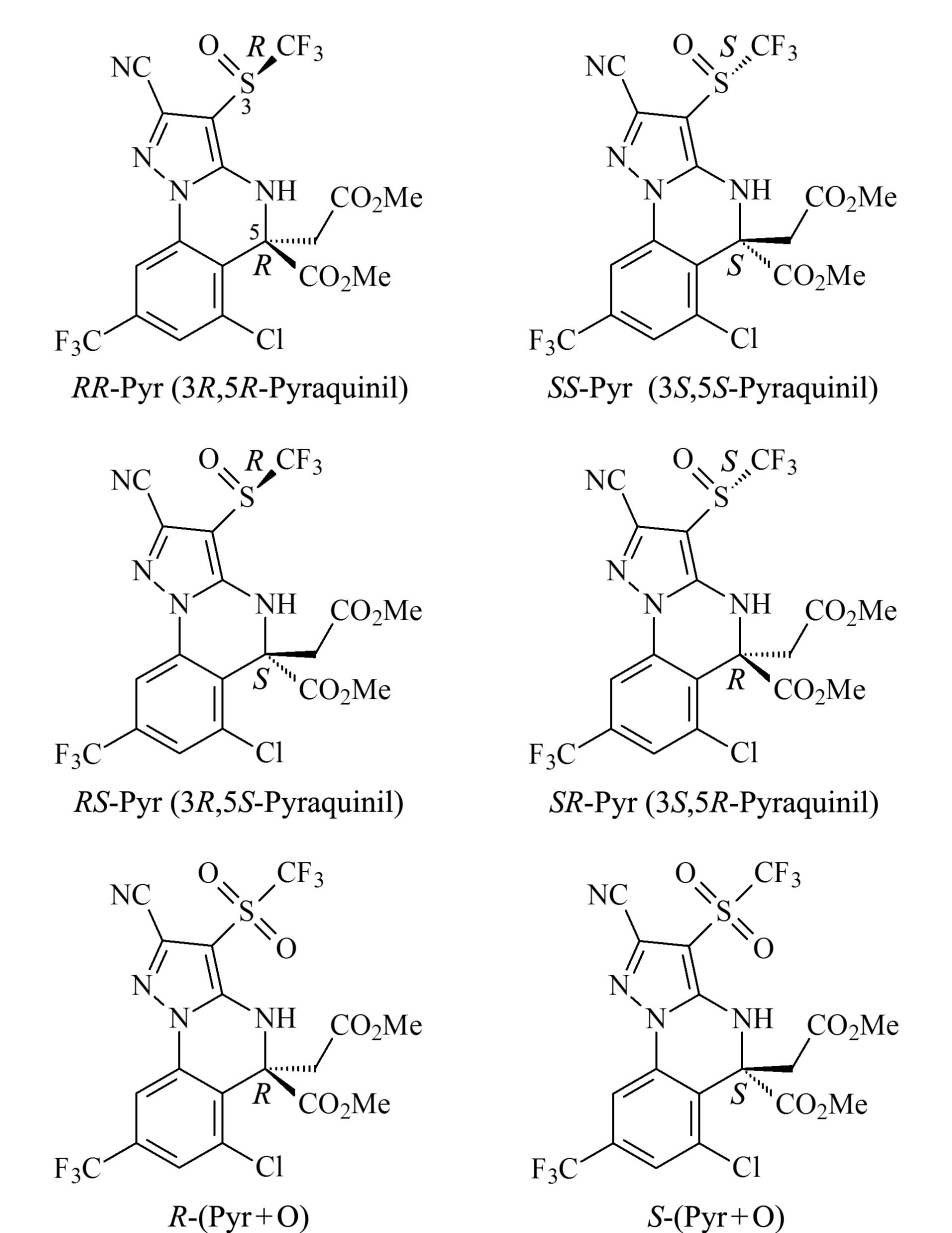
唑虫酯及其氧化代谢产物异构体的绝对构型

目前关于唑虫酯的相关研究都是基于外消旋体来进行的,并未区分手性对映体的区别,因此有必要建立唑虫酯对映体分离分析方法。鉴于此,本工作利用高效液相色谱-串联质谱,对唑虫酯及唑虫酯氧化代谢产物(Pyr+O)异构体进行手性拆分,并确定各异构体在色谱柱上的流出顺序。同时,建立了唑虫酯及唑虫酯氧化代谢产物异构体在蔬菜中的残留分析方法。旨在为开发和使用高效低毒的唑虫酯手性单体提供基础数据,为指导新农药的合理使用提供技术支撑,为对其环境行为研究、后续质量控制及药效评价等提供相应的分析技术。

## 1 实验部分

### 1.1 仪器、试剂与材料

LCMS-8060高效液相色谱-串联质谱联用仪(日本岛津仪器有限公司); QuEChERS自动样品制备系统(Sio-6512,北京本立科技有限公司);超纯水仪(Milli-Q,美国密理博公司);电子天平(EM204,梅特勒-托利多仪器有限公司);超声波清洗器(KQ-800KDE,昆山超声仪器有限公司);旋涡混合器(GL-88B,其林贝尔仪器制造有限公司)。

唑虫酯(纯度98%)及其氧化产物原药,来源于华南农业大学天然农药与化学生物学教育部重点实验室;甲醇、乙腈(色谱纯,德国默克集团);微孔滤膜(0.22 μm,天津津腾实验设备有限公司);甲酸铵(纯度≥99%,上海阿拉丁生化科技有限公司)。石墨化炭黑(GCB)和*N*-丙基乙二胺固相吸附剂(PSA)(200~400目,博纳艾杰尔科技);无水硫酸镁(分析纯,广州化学试剂厂)。

供试小白菜和蕹菜品种分别为甜脆小白菜8933和青梗柳叶空心菜,均采自广东省农业科学院白云基地。

### 1.2 样品提取与净化

称取10 g(精确至0.01 g)蔬菜样品置于50 mL离心管中,加入20 mL乙腈,并加入研磨珠,涡旋振荡30 s;再加入1.0 g氯化钠、4.0 g无水硫酸镁涡旋混匀,离心振荡一体机以1000 r/min速度振荡5 min,以4000 r/min速度离心5 min。取8.0 mL上清液于装有1.0 g净化剂(0.835 g MgSO_4_+0.150 g PSA+0.015 g GCB)的离心管中,以1000 r/min速度振荡2 min,以4000 r/min速度离心2 min,取上清液,过0.22 μm的有机滤膜,待测。

### 1.3 标准溶液配制及标准曲线绘制

分别称取250.0 mg外消旋唑虫酯和氧化产物标准品至250 mL棕色容量瓶中,用甲醇溶解,配制成1000 mg/L的母液。将上述母液用乙腈逐级稀释成质量浓度分别为1、5、10、50、100、500和2000 μg/L的标准工作溶液备用。称取空白样品,按1.2节的方法前处理后,获得空白基质液,分别用基质配制质量浓度为5、10、50、100、500、1000和5000 μg/L的基质标准工作溶液。以标准溶液峰面积平均值(*Y*)与标准溶液质量浓度(*X*, μg/L)进行线性回归,绘制基质标准曲线。

### 1.4 色谱-质谱条件

#### 1.4.1 色谱条件

纤维素-三(3,5-二氯苯基氨基甲酸酯)共价键键合手性色谱柱(Chiral INC, 250 mm×4.6 mm, 5 μm,购自广州菲罗门科学仪器有限公司);流动相A相为2 mmol/L甲酸铵水溶液,B相为乙腈。梯度洗脱程序:0~2.5 min, 43%B; 2.5~57 min, 43%B~46%B; 57~58 min, 46%B~43%B; 58~60 min, 43%B。柱温:28 ℃;进样量:1 μL;流速:0.5 mL/min。

#### 1.4.2 质谱条件

电喷雾离子源(ESI),负离子扫描;加热模块温度:400 ℃;脱溶剂温度:250 ℃;雾化气流量:3.0 L/min;干燥气流量:10 L/min;检测方式:多反应监测(MRM);驻留时间:0.03 s。其他参数见表1。

**表 1:** 唑虫酯及其代谢产物的质谱参数

Compound	Parent ion (m/z)	Product ion (m/z)	Dwell time/ms	Q1 voltage/V	Collision energy/eV	Q3 voltage/V
Pyr	543.00	349.00^*^	30.0	24.0	39.0	23.0
		363.00	30.0	24.0	36.0	24.0
Pyr+O	559.00	352.90^*^	30.0	22.0	25.0	17.0
		366.90	30.0	26.0	35.0	22.0

*Quantitative ion.

### 1.5 数据处理

#### 1.5.1 分离度计算

分离度(*R*_s_)的计算方法见公式(1):


(1) $R_{\mathrm{s}}=\frac{2\left(t_{2}-t_{1}\right)}{w_{1}+w_{2}}$

其中,*t*为保留时间,*w*为峰宽。

#### 1.5.2 基质效应

基质效应(matrix effects, ME)是指共流出物影响分析物的离子化效率,使其分析信号增强或减弱的现象。计算基质效应的公式如下:


(2) $\mathrm{ME}=\frac{k_{2}-k_{1}}{k_{1}} \times 100 \%$

其中,*k*_2_代表基质匹配标准曲线的斜率,*k*_1_代表溶剂标准曲线的斜率。当ME为正值时为基质增强效应,当ME为负值时为基质减弱效应。基质效应在-20%~20%之间为弱基质效应;在-50%~-20%和20%~50%之间为中等基质效应;超过-50%或50%为强基质效应^[26]^。

## 2 结果与讨论

### 2.1 唑虫酯异构体拆分条件优化

#### 2.1.1 不同手性固定相对唑虫酯异构体分离的影响

手性柱中的固定相对化合物的手性分离往往起决定性作用。目前市场上用于色谱分离的手性固定相大致分为多糖(cellulose)型、环糊精(cyclodextrin)型、大环抗生素(macrocyclic antibiotics)型、蛋白质(protein)型以及冠醚(crown enters)型等。其中,多糖型是商品化手性固定相中应用最广泛的类型之一,包括纤维素型和淀粉型^[27]^。多糖是一类具有天然光学活性的高分子化合物,其葡萄糖单元上的羟基易于被修饰和衍生化,表现出特殊的手性识别能力^[28]^。因此本研究选择6种多糖型的手性柱,在日本岛津LC-20A高效液相色谱仪上比较了不同手性柱对唑虫酯异构体的分离效果。6种手性分离色谱柱:直链淀粉-三(3,5-二甲基苯基氨基甲酸酯)(Chiral ND(2)-RH)、直链淀粉-三(5-氯-2-甲基苯基氨基甲酸酯)(Chiral NY(2)-RH)、直链淀粉-三(4-氯-3-甲基苯基氨基甲酸酯)(Chiral NX(2)-RH)、纤维素-三(4-氯-3-甲基苯基氨基甲酸酯)(Chiral MX(2)-RH)、纤维素-三(3,5-二氯苯基氨基甲酸酯)共价键键合(Chiral INC)和纤维素-三(3,5-二甲基苯基氨基甲酸酯)共价键键合(Chiral INB);以上手性柱均购自广州菲罗门科学仪器有限公司,规格为250 mm×4.6 mm, 5 μm。

结果如图2所示,在所测试的手性固定相中,Chiral ND(2)-RH柱无法分离唑虫酯异构体,Chiral NY(2)-RH柱和Chiral NX(2)-RH柱只能分离出2种异构体,Chiral MX(2)-RH可分离3种异构体,Chiral INB柱未能完全分离4种异构体,而Chiral INC手性柱成功实现了唑虫酯4个异构体的基线分离,表现出最佳的拆分性能。

**图 2:**
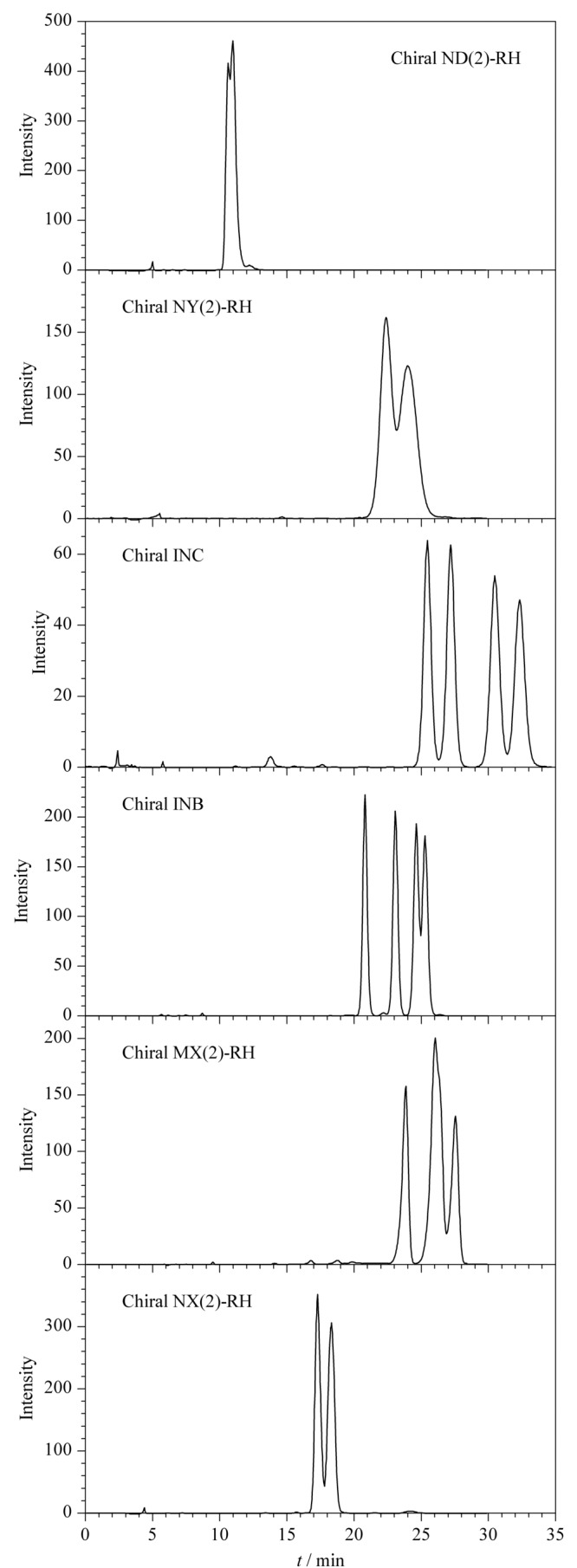
不同手性柱分离唑虫酯异构体的色谱图

另外,根据文献报道,纤维素类手性柱与直链淀粉类手性柱的手性识别能力存在很大差别,且纤维素类手性柱的稳定性更高^[27]^,键合型纤维素类手性固定相可以通过流动相的优化来实现很多在常规流动相中难以分离的对映体的拆分^[28]^。综合考虑,本工作选择键合型纤维素类固定相的Chiral INC手性柱作为唑虫酯异构体的拆分柱。

#### 2.1.2 流动相比例对唑虫酯及其氧化产物异构体分离的影响

流动相的选择和优化对于目标物质的分离起着至关重要的作用,本工作以2 mmol/L甲酸铵水溶液和乙腈为流动相,设置6个洗脱程序,分别计算不同洗脱条件下唑虫酯及其氧化产物异构体两个色谱峰之间的分离度(见表2),比较不同流动相比例下唑虫酯及其氧化产物的拆分效果。

**表 2:** 不同洗脱条件下唑虫酯及其氧化产物异构体的分离度

Elution condition number	φ(Mobile phase B)/%	Acquisition time/min	R_s1_	R_s2_	R_s3_	R_s4_
1	20-95-95-20	5-35-40-45	0.53	1.37	0.96	2.93
2	40-95-95-40-40	5-50-65-70-80	1.03	2.05	1.31	3.88
3	40-70-95-95-40-40	5-35-40-45-48-50	1.12	2.10	1.34	3.97
4	42-55-95-95-42-42	4-55-58-62-65-70	1.62	2.64	1.62	5.47
5	42-48-42-42	2.5-66-67-70	1.63	2.81	1.78	6.03
6	43-46-43-43	2.5-57-58-60	1.63	2.83	1.74	5.82

Mobile phase B: acetonitrile; *R*_s1_: resolution of peaks 1 and 2 in Fig. 3; *R*_s2_: resolution of peaks 2 and 3 in Fig. 3; *R*_s3_: resolution of peaks 3 and 4 in Fig. 3; *R*_s4_: resolution of peaks 5 and 6 in Fig. 3.

从表2可知,唑虫酯及氧化产物异构体分离度随有机相初始比例的提高而变大,同时受提高速度的影响,提高速度越慢,分离度越大。在洗脱程序1~3条件下,唑虫酯各异构体之间分离度为0.53~2.10,氧化产物分离度为2.93~3.97;洗脱程序4~6条件下,唑虫酯各异构体之间的分离度为1.62~2.83。通常,当分离度高于1.5时就认为各色谱峰之间实现了基线分离。

因此,洗脱程序1~3条件下,目标物质的各异构体之间难以完全分离;洗脱程序4~6条件下,目标物质的各异构体之间实现基线分离。由于在洗脱程序6条件下完全分离所需分析时间为60 min,短于洗脱程序4~5条件下完全分离所需分析时间70 min,综合考虑唑虫酯异构体分离参数(分离度高和分析时间短),本研究确定洗脱程序6(即1.4.1节梯度程序)为唑虫酯及其氧化产物异构体在HPLC-MS/MS上的最佳分离条件,如图3所示,唑虫酯4个异构体和氧化产物2个异构体均得到完全分离。

**图 3:**
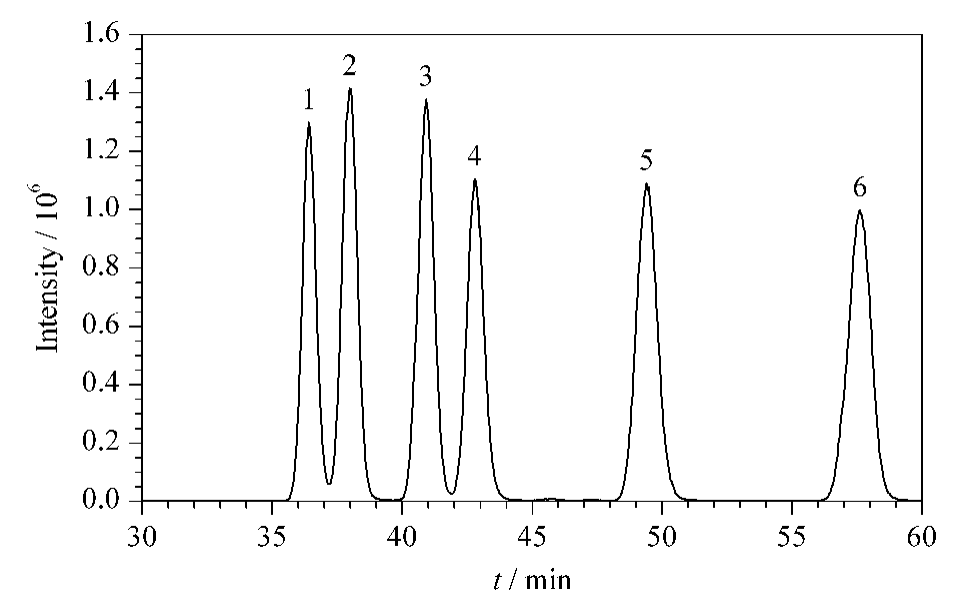
采用表2中洗脱程序6时唑虫酯及唑虫酯氧化产物的分离色谱图

#### 2.1.3 唑虫酯及其氧化产物手性异构体的出峰顺序

前期通过对唑虫酯4个手性异构单体进行单晶培养,确认了*RS*、*SR*手性异构体的绝对构型。本研究对唑虫酯4个手性异构体进行衍生化,可以得知*RS*-Pyr和*SS*-Pyr氧化之后将得到相同的产物,即*S*-(Pyr+O),而*RR*-Pyr和*SR*-Pyr氧化之后将得到相同的产物,即*R*-(Pyr+O)。因此对单晶结果确认的*RS*、*SR*手性异构体氧化之后分别纯化,获得了*S*型和*R*型唑虫酯氧化产物的两个手性单体的色谱图(见图4b, 4e),两个手性单体的出峰顺序为*S*-(Pyr+O)、*R*-(Pyr+O),如图4a所示。再根据确认的*S*型和*R*型唑虫酯氧化产物反向确认*SS*-Pyr和*RR*-Pyr,如图4c和4d。

**图 4:**
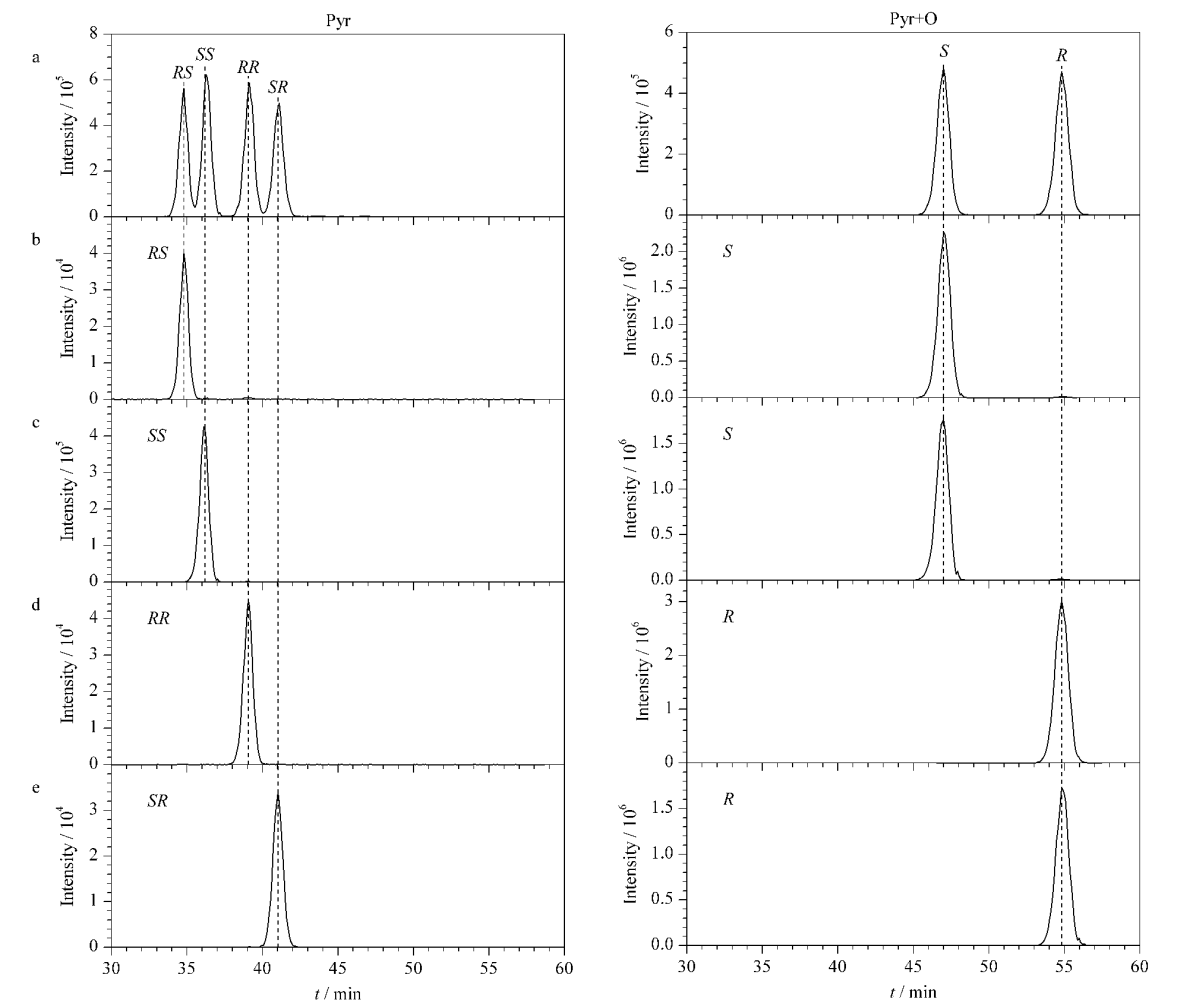
唑虫酯及唑虫酯氧化产物异构体在手性柱Chiral INC上的出峰顺序

综上所述,唑虫酯4个手性异构体依次出峰顺序为*RS*-Pyr、*SS*-Pyr、*RR*-Pyr和*SR*-Pyr;唑虫酯氧化产物异构体出峰顺序为*S*-(Pyr+O)和*R*-(Pyr+O)(见图4a)。

### 2.2 前处理净化条件的优化

QuEChERS前处理法是目前广泛应用的前处理方法^[29]^。鉴于此,本研究参考GB 23200.113-2018中蔬菜的QuEChERS前处理方法,综合比较3种不同组合(0.850 g MgSO_4_+0.150 g PSA、0.985 g MgSO_4_+0.015 g GCB和0.835 g MgSO_4_+0.150 g PSA+0.015 g GCB)的净化剂对唑虫酯及其氧化产物不同异构体在蔬菜中回收率的影响(见图5)。由图5可以看出,对于唑虫酯及其氧化产物6个异构体而言,0.850 g MgSO_4_+0.150 g PSA和0.835 g MgSO_4_+0.150 g PSA+0.015 g GCB为净化剂时,回收率在80%~110%之间;0.985 g MgSO_4_+0.015 g GCB为净化剂时,在蕹菜中,*R*-(Pyr+O)和*S*-(Pyr+O)的回收率小于80%,在小白菜中唑虫酯及其氧化产物6个异构体回收率均大于110%。由于GCB主要对色素具有一定的吸附作用,蕹菜和小白菜属于颜色较深的蔬菜,因此综合考虑,选择0.835 g MgSO_4_+0.150 g PSA+0.015 g GCB作为最优净化剂组合。

**图 5:**
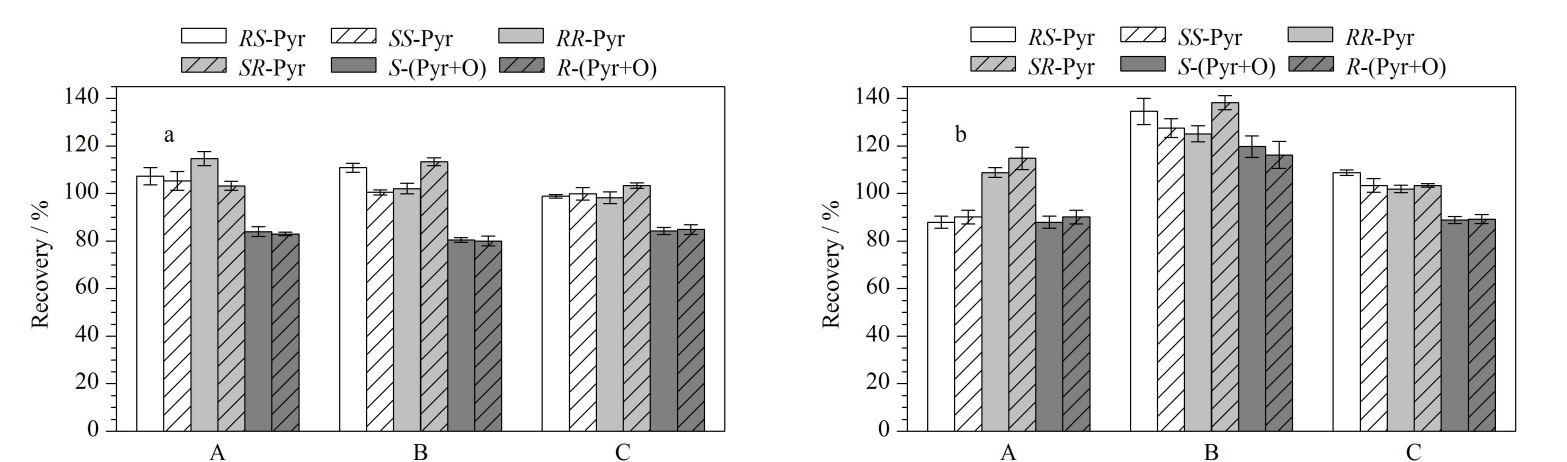
唑虫酯及其氧化产物异构体在(a)蕹菜和(b)小白菜中的回收率(*n*=3)

### 2.3 方法学考察

#### 2.3.1 基质效应

本研究采用公式(2)的方法评价唑虫酯及其氧化代谢产物异构体在不同蔬菜中的ME。从表3可看出,唑虫酯及其氧化代谢产物异构体的ME在0.7%~30.6%之间,均为正值。因此在蕹菜和小白菜中均表现出基质增强效应。其中*SS*-Pyr、*RR*-Pyr、*R*-(Pyr+O)和*S*-(Pyr+O)在小白菜和蕹菜中均表现出弱基质效应;*RS*-Pyr在小白菜中表现出弱基质效应,在蕹菜中表现出中等基质效应;*SR*-Pyr在蕹菜和小白菜中表现出中等基质效应。由于不同异构体在不同基质中的ME大小不一,本工作为确保结果的准确性,采用基质标准溶液为基准进行校正。

**表 3:** 唑虫酯及其氧化产物异构体的线性关系和基质效应

Analyte	Matrix	Linear range/(μg/L)	Standard curve	R^2^	Matrix effect/%
RS-Pyr	acetonitrile	0.25-500	Y=8.7×10^7^X-1.6×10^4^	0.9999	
	water spinach	1.25-1250	Y=1.1×10^8^X+4.3×10^5^	0.9998	26.8
	pakchoi	1.25-1250	Y=9.3×10^7^X+1.3×10^5^	0.9999	6.1
SS-Pyr	acetonitrile	0.25-500	Y=1.1×10^8^X-2.6×10^3^	0.9999	
	water spinach	1.25-1250	Y=1.1×10^8^X+5.1×10^5^	0.9998	1.9
	pakchoi	1.25-1250	Y=1.2×10^8^X+1.6×10^6^	0.9987	10.2
RR-Pyr	acetonitrile	0.25-500	Y=1.1×10^8^X+3.4×10^4^	0.9999	
	water spinach	1.25-1250	Y=1.1×10^8^X+5.0×10^5^	0.9998	0.7
	pakchoi	1.25-1250	Y=1.2×10^8^X+1.6×10^6^	0.9987	9.0
SR-Pyr	acetonitrile	0.25-500	Y=9.2×10^7^X+1.4×10^4^	0.9999	
	water spinach	1.25-1250	Y=1.1×10^8^X+4.4×10^5^	0.9998	20.7
	pakchoi	1.25-1250	Y=1.2×10^8^X+1.4×10^6^	0.9988	30.6
S-(Pyr+O)	acetonitrile	0.5-100	Y=6.5×10^7^X+2.1×10^4^	0.9999	
	water spinach	2.5-2500	Y=6.9×10^7^X+8.3×10^5^	0.9996	7.3
	pakchoi	2.5-2500	Y=7.6×10^7^X+2.2×10^6^	0.9981	17.1
R-(Pyr+O)	acetonitrile	0.5-100	Y=6.6×10^7^X+2.7×10^4^	0.9999	
	water spinach	2.5-2500	Y=7.2×10^7^X+6.9×10^5^	0.9997	9.0
	pakchoi	2.5-2500	Y=7.8×10^7^X+2.0×10^6^	0.9999	18.2

Y: peak area; X: mass concentration, μg/L; R^2^: correlation coefficient.

#### 2.3.2 线性范围和定量限

当唑虫酯的手性异构体质量浓度在1.25~1250 μg/L之间及唑虫酯氧化代谢产物手性异构体质量浓度在2.5~2500 μg/L之间时,基质标准溶液峰面积与对应质量浓度呈现良好的线性关系,标准曲线和相关系数如表3所示。以满足农残分析回收率要求的最低加标水平为方法定量限(LOQ),唑虫酯异构体的LOQ为0.25 μg/kg,唑虫酯氧化代谢产物手性异构体的LOQ为0.5 μg/kg。

#### 2.3.3 准确度和精密度

在蕹菜和小白菜样品中同时添加唑虫酯和唑虫酯氧化产物消旋体进行添加回收试验,添加水平为1、20、400 μg/kg,此时样品中唑虫酯异构体对应添加水平为0.25、5、100 μg/kg,唑虫酯氧化代谢产物异构体对应添加水平为0.5、10、200 μg/kg,在3天内每天添加一次,每个水平做3个平行,按本文方法进行测定。通过比较同一天内目标物加标回收率的相对标准偏差(RSD)来考察日内重复性,通过分析3天内目标物回收率的RSD来考察日间重复性。

由表4可知,蕹菜中3个加标水平下该方法的平均回收率为72.6%~109.9%,日内重复性RSD为0.5%~8.7%;日间重复性RSD为1.0%~8.6%。由表5可知,小白菜中3个加标水平下该方法的平均回收率为81.2%~110.6%,日内RSD为0.6%~8.3%;日间RSD为1.1%~7.6%。实验结果表明,该方法的回收率和精密度符合定量分析要求,适用于蕹菜和小白菜中唑虫酯及其氧化产物异构体的分析。

**表 4:** 唑虫酯及氧化产物异构体在蕹菜中的加标回收率

Analyte	Spiked level/ (μg/kg)	Intra-day (n=3)								Inter-day RSD/% (n=9)
		Day 1			Day 2			Day 3		
		Recovery/%	RSD/%		Recovery/%	RSD/%		Recovery/%	RSD/%	
RS-Pyr	0.25	101.1	1.6		105.7	5.0		96.3	5.1	4.6
	5	98.8	1.2		104.9	2.2		102.8	7.2	3.0
	100	107.8	1.8		108.5	2.0		105.5	3.6	1.5
SS-Pyr	0.25	100.0	9.4		96.5	1.9		90.2	1.4	5.2
	5	99.9	4.5		94.5	3.0		94.2	8.0	3.3
	100	103.6	0.7		99.5	2.0		95.3	2.7	4.2
RR-Pyr	0.25	104.1	6.0		105.8	8.7		101.1	3.2	2.3
	5	98.2	4.4		95.4	3.2		97.1	7.6	1.5
	100	109.5	1.4		97.6	1.2		98.1	1.2	6.6
SR-Pyr	0.25	108.8	8.3		100.9	6.6		108.2	8.0	4.1
	5	103.4	1.9		105.0	1.7		109.9	6.1	3.2
	100	108.3	0.7		106.6	2.5		106.3	1.8	1.0
S-(Pyr+O)	0.5	72.6	5.2		83.3	2.0		76.4	2.7	7.0
	10	84.3	3.2		88.1	4.5		89.2	2.9	3.0
	200	88.1	2.4		91.2	0.6		90.6	0.8	1.8
R-(Pyr+O)	0.5	91.7	8.4		83.9	3.5		77.1	2.7	8.6
	10	84.9	4.3		80.9	3.4		83.6	2.2	2.4
	200	88.7	2.0		83.3	0.5		92.8	1.6	5.4

**表 5:** 唑虫酯及氧化产物异构体在小白菜中的加标回收率

Analyte	Spiked level/ (μg/kg)	Intra-day (n=3)								Inter-day RSD/% (n=9)
		Day 1			Day 2			Day 3		
		Recovery/%	RSD/%		Recovery/%	RSD/%		Recovery/%	RSD/%	
RS-Pyr	0.25	108.1	8.3		99.4	4.7		109.8	2.1	5.3
	5	108.8	3.3		106.4	1.1		109.6	0.9	1.5
	100	108.6	2.8		102.5	2.0		97.3	3.9	5.5
SS-Pyr	0.25	106.4	5.0		103.2	7.8		98.7	2.4	3.8
	5	101.5	3.4		106.1	0.7		99.6	2.6	3.3
	100	103.5	2.6		98.6	1.9		93.1	7.7	5.3
RR-Pyr	0.25	97.0	7.1		97.2	6.1		102.7	3.6	3.3
	5	105.0	7.4		110.3	2.0		110.6	1.3	2.9
	100	105.0	2.1		100.4	1.2		96.6	7.8	4.2
SR-Pyr	0.25	97.1	7.7		106.8	2.2		104.7	3.1	4.9
	5	105.8	7.1		109.6	1.4		107.1	0.6	1.8
	100	106.9	1.9		107.0	4.5		103.6	6.2	1.8
S-(Pyr+O)	0.5	94.9	4.4		82.5	7.9		85.0	3.4	7.5
	10	88.0	5.1		86.9	2.5		88.8	1.9	1.1
	200	88.7	0.8		81.2	1.9		87.9	5.1	4.8
R-(Pyr+O)	0.5	100.1	2.6		88.5	3.3		87.5	3.1	7.6
	10	90.1	5.4		85.7	1.4		88.0	1.1	2.5
	200	90.9	1.0		87.0	2.0		92.0	5.7	2.9

## 3 结论

通过对影响高效液相色谱分离分析的主要因素反相手性色谱柱和流动相等参数进行优化,在高效液相色谱-质谱联用仪上建立了拆分唑虫酯及其氧化代谢产物异构体的方法,并建立了蕹菜和小白菜中残留分析的方法,方法的准确度和精密度满足农残检测要求;同时利用衍生化的方法确定了唑虫酯以及唑虫酯氧化代谢产物异构体的出峰顺序,在Chiral INC反相色谱柱上的出峰顺序依次为*RS*-、*SS*-、*RR*-、*SR*-唑虫酯、*S*-和*R*-唑虫酯氧化产物。本研究可为新型手性农药唑虫酯的环境选择性行为研究、质量控制及药效评价等方面提供相应技术支持,同时也为新农药开发及应用提供有力的理论支撑。
